# Cell Derived Matrix Fibulin-1 Associates With Epidermal Growth Factor Receptor to Inhibit Its Activation, Localization and Function in Lung Cancer Calu-1 Cells

**DOI:** 10.3389/fcell.2020.00522

**Published:** 2020-07-03

**Authors:** Keerthi Harikrishnan, Omkar Joshi, Saili Madangirikar, Nagaraj Balasubramanian

**Affiliations:** Indian Institute of Science Education and Research, Pune, India

**Keywords:** FBLN1, EGFR, ECM, cell derived matrix, matrix microenvironment, lung cancer

## Abstract

Epidermal Growth Factor Receptor (EGFR) is a known promoter of tumor progression and is overexpressed in lung cancers. Growth factor receptors (including EGFR) are known to interact with extracellular matrix (ECM) proteins, which regulate their activation and function. Fibulin-1 (FBLN1) is a major component of the ECM in lung tissue, and its levels are known to be downregulated in non-small cell lung cancers (NSCLC). To test the possible role FBLN1 isoforms could have in regulating EGFR signaling and function in lung cancer, we performed siRNA mediated knockdown of FBLN1C and FBLN1D in NSCLC Calu-1 cells. Their loss significantly increased basal (with serum) and EGF (Epidermal Growth Factor) mediated EGFR activation without affecting net EGFR levels. Overexpression of FBLN1C and FBLN1D also inhibits EGFR activation confirming their regulatory crosstalk. Loss of FBLN1C and FBLN1D promotes EGFR-dependent cell migration, inhibited upon Erlotinib treatment. Mechanistically, both FBLN1 isoforms interact with EGFR, their association not dependent on its activation. Notably, cell-derived matrix (CDM) enriched FBLN1 binds EGFR. Calu-1 cells plated on CDM derived from FBLN1C and FBLN1D knockdown cells show a significant increase in EGF mediated EGFR activation. This promotes cell adhesion and spreading with active EGFR enriched at membrane ruffles. Both adhesion and spreading on CDMs is significantly reduced by Erlotinib treatment. Together, these findings show FBLN1C/1D, as part of the ECM, can bind and regulate EGFR activation and function in NSCLC Calu-1 cells. They further highlight the role tumor ECM composition could have in influencing EGFR dependent lung cancers.

## Highlights

– FBLN1C/1D suppress EGFR activation and EGFR dependent migration of Calu-1 cells.
– FBLN1C/1D isoforms bind EGFR, independent of its activation.
– Cell derived matrix FBLN1 associates with EGFR in Calu-1 cells.
– Cell derived matrix FBLN1C and FBLN1D regulate EGFR activation and localization to regulate cell adhesion, spreading in Calu-1 cells.

## Introduction

Lung cancer is the leading cause of cancer related deaths worldwide. Non-small cell lung cancer (NSCLC) accounts for 85% of the cases (Chen et al., 2014). Epidermal growth factor receptor (EGFR) is a protein that is expressed on the cell surface and influences cell growth, survival and motility (Normanno et al., 2006). EGFR is overexpressed in lung cancer (Sharma et al., 2007; Malik and Raina, 2015) and is associated with poor prognosis (Gridelli et al., 2003; Scagliotti et al., 2004). Tumors that are initially responsive to EGFR targeted therapies can also acquire drug resistance rendering their treatment ineffective (Gridelli et al., 2003; Wheeler et al., 2010). The extracellular matrix (ECM), as a vital regulatory player in the tumor microenvironment not only provides structural support but also regulates downstream signaling to control cell growth, survival, differentiation and motility (Lu et al., 2012; Pickup et al., 2014). Crosstalk between the tumor ECM and growth factor receptors (like EGFR) has been further shown to play an important functional role in mediating tumor progression and metastasis (Wu et al., 2004; Reticker-Flynn et al., 2012; Du et al., 2013; Kim et al., 2014; Stevens et al., 2017). In the lungs, proteomic analysis shows ECM proteins to be a major component of the cellular microenvironment (Burgstaller et al., 2017). FBLN1 is prominently expressed in lung ECM, significantly more than other FBLN isoforms (Consortium, 2015; Burgstaller et al., 2017; Krasny et al., 2018). Although a large number of matrix proteins are altered in lung cancers, the functional role of some of the major lung ECM proteins like Fibulin-1, Elastin, Nephronectin, Agrin, Laminin remain poorly documented (Burgstaller et al., 2017; Gocheva et al., 2017).

Fibulins are a family of secreted glycoproteins that consist of a series of epidermal growth factor (EGF) like repeats followed by a C terminal fibulin type module (Argraves et al., 1990). Fibulin-1 is the prototypic member of this family of proteins, and is highly expressed in blood vessels, skin, heart, and lung (Argraves et al., 2003; Chu and Tsuda, 2004; Lau et al., 2010). Alternative splicing of FBLN1 produces two splice variants in mice (FBLN1C/FBLN1D) and four splice variants in humans (FBLN1A/B/C/D) (Argraves et al., 2003). Human FBLN1A/1B are known to be restricted to the placenta making human FBLN1C/1D the prevalent isoforms in human tissues (Tran et al., 1997). FBLN1 has a diverse array of ligands and has been shown to interact with other ECM proteins including Versican (Aspberg et al., 1999), Aggrecan (Aspberg et al., 1999), Laminin (Sasaki et al., 1995; Timpl et al., 2000; Ogawa et al., 2007), Tropoelastin (Sasaki et al., 1999), Nidogen (Sasaki et al., 1995), and Fibronectin (Balbona et al., 1992). FBLN1 also interacts with growth factors including heparin-binding epidermal growth factor (HB-EGF) (Brooke et al., 2002), connective tissue growth factor (CCN2) and CCN3 (Notch ligand) (Perbal et al., 1999). The significance of these interactions in regulating cellular processes is only beginning to be understood. FBLN1C and FBLN1D isoforms also have distinct biological roles based on their differential affinity and localization with other matrix proteins (Sasaki et al., 1995; Perbal et al., 1999; Brooke et al., 2002; Muriel et al., 2006).

Fibulin-1 is seen to exhibit both pro-oncogenic as well as tumor suppressive effects (Gallagher et al., 2005). FBLN1 is upregulated in breast and ovarian cancers where FBLN1C is expressed at higher levels than FBLN1D (Moll et al., 2002; Bardin et al., 2005). As a tumor suppressor FBLN1 levels are downregulated in epithelial cancers including melanoma (Wu et al., 2014), squamous cell carcinoma (Zhang et al., 2013), renal cell carcinoma (Xiao et al., 2013), hepatocellular carcinoma (Kanda et al., 2011), gastric carcinoma (Cheng et al., 2008), prostate carcinoma (Wlazlinski et al., 2007), colorectal carcinoma (Pesson et al., 2014), and lung adenocarcinoma (LUAD) (Cui et al., 2015). Further, overexpression of FBLN1D in fibrosarcoma cells is seen to inhibit tumor growth *in vivo* (Qing et al., 1997), with purified placental FBLN1 seen to inhibit adhesion, spreading, motility and invasion of breast cancer cells (Twal et al., 2001). Recent studies show that FBLN1 levels are downregulated in patients with NSCLC and is associated with poor prognosis (Yue et al., 2009; Cui et al., 2015).

Extracellular matrix proteins have been known to sequester growth factors (Grahovac and Wells, 2014; Hastings et al., 2019) and growth factor receptors to regulate their function (Kim et al., 2011; Grahovac and Wells, 2014). An important example of this is Fibronectin binding to VEGF and its regulation of angiogenesis (Zhu and Clark, 2014). In cancers, ECM proteins like Perlecan, Versican, Aggrecan, Decorin and Biglycan all bind growth factors to support pro-tumorigenic as well as anti-tumorigenic effects (Hynes and Naba, 2012; Grahovac and Wells, 2014). ECM proteins Laminin-5 (Schenk et al., 2003), Tenascin-C (Iyer et al., 2008), and Decorin (Iozzo et al., 1999) bind EGFR to regulate its activation and function in cancers (Grahovac and Wells, 2014).

Epidermal growth factor receptor activation is a vital regulator of oncogenic signaling in cancer cell invasion and metastasis (Normanno et al., 2006). EGFR is overexpressed in a variety of epithelial carcinomas, including neuronal, breast, and lung (Normanno et al., 2006; Bakker et al., 2017). It is subject to multiple regulatory cues including the ECM (Kim et al., 2011; Stevens et al., 2017). ECM composition is altered dynamically during cancer progression (Lu et al., 2012; Pickup et al., 2014), making the regulation of EGFR signaling by matrix proteins in cancers of direct interest. When compared across tissues, FBLN1 expression levels are seen to be prominent in the Lung (Consortium, 2015). Lung tissue matrisome studies have also confirmed FBLN1 to be significantly enriched (Krasny et al., 2018) making it an important candidate in ECM function. ECM-mediated EGFR signaling has been shown to regulate cell adhesion and motility (Alexi et al., 2011; Bakker et al., 2017) supporting tumorigenesis (Grahovac and Wells, 2014). Tenascin-C mediated EGFR activation drives cell migration and invasion in melanomas (Shao et al., 2015). Versican, Thrombospondin -1 and SPARC can all regulate EGFR activation, though their direct association is not known (Grahovac and Wells, 2014). FBLN3 binds EGFR through its EGF like repeats inhibiting its activation and function in lung and brain cancer cells (Kim et al., 2014; Wang et al., 2017).

Changes in lung tumor ECM have been shown to affect growth factor signaling pathways regulating EMT, cell proliferation, survival and migration to drive oncogenic transformation (Rintoul and Sethi, 2001; Pirinen et al., 2005; Lim et al., 2017). Cell-derived matrices (CDM) in better representing the tumor ECM composition elicit a more physiological tumor cell response (Scherzer et al., 2015; Kaukonen et al., 2017). This study reveals the role FBLN1 isoforms FBLN1C and FBLN1D as part of the CDM have in regulating EGFR activation and function in lung cancer cells.

## Results

### Fibulin-1 Levels Are Significantly Downregulated in Lung Cancer

**FIGURE 1:**
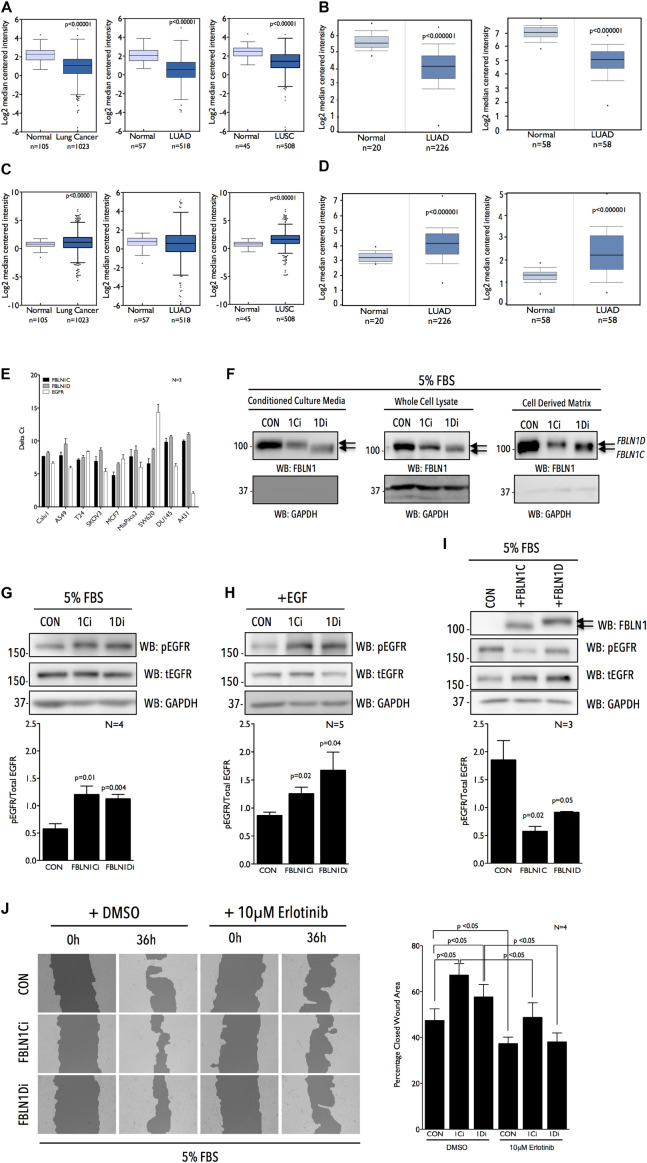
Fibulin-1 is a negative regulator of EGFR activation and function in Calu-1 cells. Graphs represent transcript levels of FBLN1 **(A,B)** and EGFR **(C,D)** in normal lung and pan lung cancer, lung adenocarcinoma (LUAD) and lung squamous cell carcinoma (LUSC) tissues from TCGA database **(A,C)** and normal lung and lung adenocarcinoma (LUAD) tissues (Okayama et al., 2012 Cancer Research and Selamat et al., 2012 Genome Research) from Oncomine database **(B,D)**. **(E)** RTPCR analysis evaluates FBLN1C (Black bar), FBLN1D (Gray bar), and EGFR (White bar) expression in a panel of cancer cell lines listed. Graph represents mean ± SE of Delta Ct values from three independent experiments. **(F)** Western blot was used to detect Fibulin-1 (WB: FBLN1) and GAPDH (WB: GAPDH) in conditioned culture media, whole cell lysate and cell derived matrix from FBLN1C (1Ci), FBLN1D (1Di) knockdown and control (CON) Calu-1 cells grown with 5% FBS. Arrows mark the position of FBLN1C and FBLN1D isoforms in the representative blots. Blot is best representative of three independent experiments. **(G–I)** Western blot detection of EGFR phosphorylated on tyrosine1173 (WB: pEGFR), total EGFR (WB: tEGFR), and GAPDH (WB: GAPDH) in lysates from Calu-1 cells **(G)** in the presence of serum growth factors (5% FBS), **(H)** on stimulation with EGF (100 ng/ml) for 5 min (+EGF) in serum deprived control (CON), FBLN1C (1Ci), FBLN1D (1Di) knockdown cells and **(I)** in the presence of serum growth factors (5% FBS) in Calu-1 cells overexpressing untagged FBLN1C or FBLN1D. Overexpression of Fibulin-1C (+FBLN1C) and Fibulin-1D (+FBLN1D) was confirmed by western blot (WB: FBLN1). Arrows mark the position of FBLN1C and FBLN1D isoforms in the representative blots. **(G–I)** Bar graphs represents mean ± SE of pEGFR to total EGFR ratio from 3 to 5 independent experiments as indicated in each graph. Statistical analysis of the data was done using the students *t*-test and *p* values are as shown. **(J)** Representative images of wound healing assay done in the presence of serum growth factor (5% FBS) in Control (CON) vs. FBLN1C (1Ci) vs. FBLN1D (1Di) knockdown Calu-1 cells at 0 h and 36 h in the presence of DMSO or 10 μM Erlotinib. Images were analyzed using T-Scratch software and the percent closed wound area calculated. Graph represents mean ± SE from four independent experiments. Statistical analysis of the data was done using two-way ANOVA and *p* values are as shown.

To test the expression of FBLN1 in lung cancers we first evaluated the TCGA lung cancer dataset (Campbell et al., 2016) and detected ∼ 2.41 fold decrease in FBLN1 transcript levels in lung cancer samples relative to normal lung (Figure 1A). NSCLC account for 85% of all lung cancers (Chen et al., 2014), with lung adenocarcinoma (LUAD) and lung squamous cell carcinoma (LUSC) being the most common pathological NSCLC subtypes. We hence looked at the LUAD and LUSC datasets in TCGA which showed a ∼2.75 and ∼2.04 fold decrease in the expression of FBLN1 relative to normal lung tissue (Figure 1A). Two LUAD datasets from Oncomine (Okayama et al., 2012; Selamat et al., 2012) also showed a ∼2.8 and ∼4.04 fold decrease in FBLN1 expression relative to normal lung tissue (Figure 1B). Together these findings confirm that FBLN1 levels are downregulated in NSCLC. The relative levels and the contribution of FBLN1C and FBLN1D isoforms in NSCLC remain untested. With EGFR overexpression in lung cancers known to be correlated with poor prognosis (Chen et al., 2014), a significant increase in EGFR transcript levels was seen in the TCGA pan lung cancer (∼1.3 fold) and LUSC (∼1.7 fold) [datasets (Figure 1C)]. The TCGA LUAD dataset showed EGFR expression to be comparable, though both the Oncomine LUAD datasets show EGFR to be overexpressed relative to normal lung tissues (∼1.89 fold) (∼2.99 fold) (Figure 1D), (Okayama et al., 2012; Selamat et al., 2012). FBLN1 and EGFR gene expression data from the TCGA Pan Lung cancer, LUAD and LUSC datasets were also compared using cBioPortal and did not show significant mutual exclusivity or co-occurrence. While such a correlation would support the presence of a functional association between FBLN1 and EGFR, the lack thereof does not preclude existence of the same.

### Fibulin-1 Isoforms Inhibit EGFR Activation and Function in Lung Cancer Calu-1 Cells

Of the four known FBLN1 isoforms relative expression of FBLN1C and 1D, known to be ubiquitously expressed, were tested across a panel of cancer cell lines by quantitative RTPCR and were found to be comparable (Figure 1E). EGFR expression did, however, vary significantly across these cell lines (Figure 1E), (Rusnak et al., 2007). NSCLC cell lines Calu-1 and A549 with comparable FBLN1C and FBLN1D expression and moderate EGFR expression were used to evaluate role of FBLN1 isoforms and their possible crosstalk with EGFR. With no commercial siRNA available for specifically targeting human FBLN1C and FBLN1D we designed siRNA to target a unique 415 bp region (EXON 18, 19, 20) in FBLN1D and 350 bp region (EXON16) in FBLN1C (Supplementary Figure 1A). This limited the number of individual siRNA sequences that showed specificity *in silico*, which were then tested *in vivo*. siRNA-mediated knockdown of FBLN1C and FBLN1D in Calu-1 cells (Supplementary Figures 1B,C) showed loss of FBLN1C did not affect FBLN1D levels significantly, and vice versa (Supplementary Figures 1B,C). The same was tested for by western blot using a total FBLN1 antibody in conditioned culture media (CCM), whole cell lysates (WCL), and cell derived matrix (CDM). FBLN1C and FBLN1D isoforms have a predicted molecular weight (MW) of ∼74 kD and ∼77 kD, respectively, but run closer to ∼100 kDa on SDS PAGE, as reported earlier (Argraves et al., 1990; Hanada and Sasaki, 2018). This could further be affected by their differential glycosylation (Argraves et al., 1990; Aspberg et al., 1999). Isoform specific siRNA mediated knockdown (Figure 1F and Supplementary Figures 1B,C) shows FBLN1D to run marginally higher on a 10% SDS PAGE as compared to FBLN1C, in CCM, WCL, and CDM (Figure 1F and Supplementary Figures 1B,C). This differential mobility of FBLN1C and 1D is further confirmed on their overexpression discussed below (Figure 1I and Supplementary Figure 1E).

We further tested if and how both Fibulin-1 isoforms regulate EGFR activation in Calu-1 cells on sustained serum growth factor stimulation. Loss of FBLN1C and FBLN1D significantly increased EGFR activation (Figure 1G and Supplementary Figure 1B) without affecting its expression (Supplementary Figure 1D). Rapid EGF stimulation (100 ng/ml) of serum deprived Calu-1 cells significantly promoted EGFR activation on loss of FBLN1C and FBLN1D (Figure 1H and Supplementary Figure 1C). Effect of FBLN1C and FBLN1D on EGFR activation was comparable (Figures 1G,H). Overexpression of untagged human FBLN1C and FBLN1D in Calu-1 cells was accordingly seen to significantly suppress EGFR activation (Figure 1I). FBLN1C overexpression had a marginally, but significantly, better effect than FBLN1D on EGFR activation. Overexpressed FBLN1 ran at ∼100 kD on 10% SDS PAGE, FBLN1D running marginally higher than FBLN1C (Figure 1I), as was seen in knockdown studies above (Figure 1F and Supplementary Figure 1E).

The effect FBLN1C/1D mediated activation of EGFR has on the migration of Calu-1 cells was tested (Normanno et al., 2006). Wound-healing assays in the presence of serum growth factors showed loss of FBLN1C and FBLN1D (Supplementary Figure 1C) to both significantly promote Calu-1 cell migration (Figure 1J and Supplementary Figure 1C). FBLN1C knockdown (as in overexpression studies) was seen to marginally better promote migration than FBLN1D, Erlotinib treatment (Supplementary Figure 1F) significantly inhibiting both their effects. Marginal differences in the relative effects of FBLN1 isoforms on EGFR activation and function could reflect their regulation to be context dependent, possibly mediated by their relative association.

We further wanted to evaluate this FBLN1-EGFR crosstalk in an additional NSCLC cell line and optimized the FBLN1C and FBLN1D knockdown in A549 cells. Quantitative RT-PCR results showed that while FBLN1C knockdown was specific (Supplementary Figure 1G), knockdown of FBLN1D was not and affected FBLN1C levels as well (Supplementary Figure 1G). We further overexpressed FBLN1C and FBLN1D to evaluate its effect on EGFR activation. Like with Calu-1 cells (Figure 1G), FBLN1C overexpression significantly inhibited EGFR activation, while FBLN1D overexpression showed a similar trend (Supplementary Figure 1H). This coupled with further studies evaluating the association of FBLN1 and EGFR in A549 cells (Supplementary Figures 3C–E) suggests this regulatory crosstalk could be conserved across NSCLC cells, though this needs further evaluation.

### Fibulin-1C and Fibulin-1D Bind EGFR, Independent of Its Activation

**FIGURE 2:**
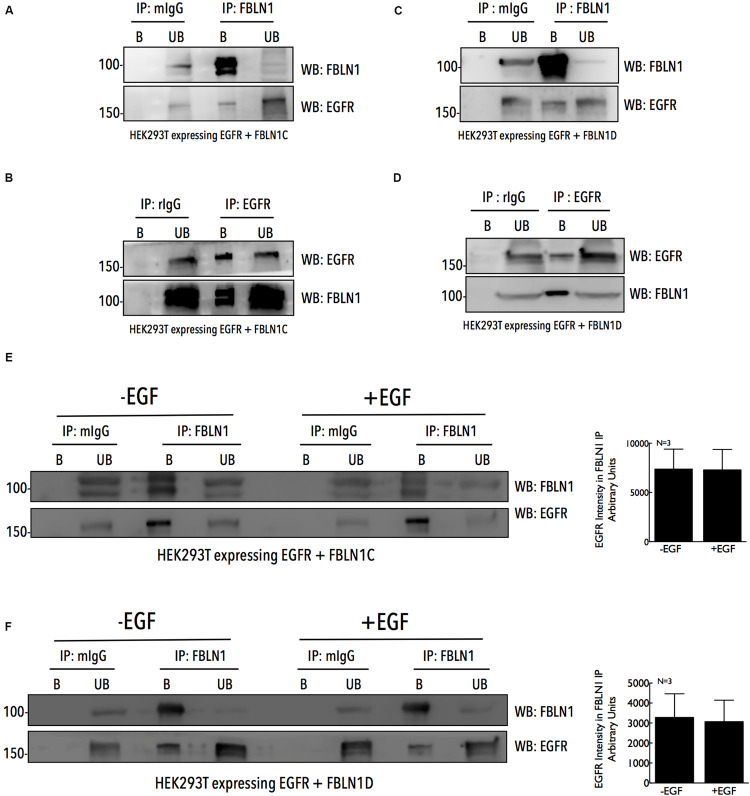
Fibulin-1C and Fibulin-1D co-immunoprecipitate EGFR, independent of its activation. Immunoprecipitated Fibulin-1 **(A,C,E,F)** (IP: FBLN1) and EGFR **(B,D)** (IP: EGFR) from HEK 293T cells, expressing EGFR-GFP **(B,D)** and untagged Fibulin-1C **(A,B)** or untagged Fibulin-1D **(C,D)** were compared to mouse IgG **(A,C,E,F)** (IP : mIgG) and rabbit IgG **(B,D)** (IP: rIgG), respectively. Immunoprecipitation of Fibulin-1 (WB: FBLN1) and EGFR (WB: EGFR) along with their co-precipitation was detected by western blot. The immunoprecipitated (bound) protein eluted and un-bound fractions (B vs. UB) were also compared by western blot. The results are representative of three independent experiments. **(E,F)** HEK 293T cells expressing untagged FBLN1C or FBLN1D and EGFR-GFP were serum starved, stimulated with EGF (100 ng/ml) for 5 min and Fibulin-1C **(E)** and Fibulin1D **(F)** immunoprecipitated. These were probed by western blot for Fibulin-1 (WB: FBLN1) (to confirm IP) and EGFR (WB: EGFR) (to detect Co-IP). The results are representative of three independent experiments that gave similar results. Bar graphs on the right represent mean ± SE of EGFR intensity detected in the FBLN1 IP with (+EGF) and without (–EGF) EGF treatment from three independent experiments as indicated.

The association of FBLN1 and EGFR could regulate its activation and function in cells, and was tested by immunoprecipitation studies. HEK293T cells overexpressing untagged FBLN1C/FBLN1D and EGFR were used to immunoprecipitate FBLN1 using anti-FBLN1 antibody and EGFR was seen to be co-immunoprecipitated (Figures 2A,C). Similarly, overexpressed EGFR immunoprecipitated with an anti-EGFR antibody co-immunoprecipitated FBLN1 (Figures 2B,D). This FBLN1-EGFR association could be direct or indirect, mediated through other proteins. Lack of FBLN1C or FBLN1D specific antibodies did not allow for the testing of their relative association with EGFR. We next tested if EGF mediated EGFR activation affects its association with FBLN1C/1D. HEK293T cells over expressing EGFR when stimulated with EGF (100 ng/ml for 5 min) increased EGFR activation (Supplementary Figures 2A,B), but did not affect its association with FBLN1C or FBLN1D (Figures 2E,F). Quantitation of EGFR intensity in FBLN1 pulldown confirms the same (Figures 2E,F). This suggests that while FBLN1 can bind and regulate EGFR activation (Figures 1G–I) its activation status does not affect their association (Figures 2E,F).

### Matrix Fibulin-1 Associates With EGFR in Calu-1 Cells

**FIGURE 3:**
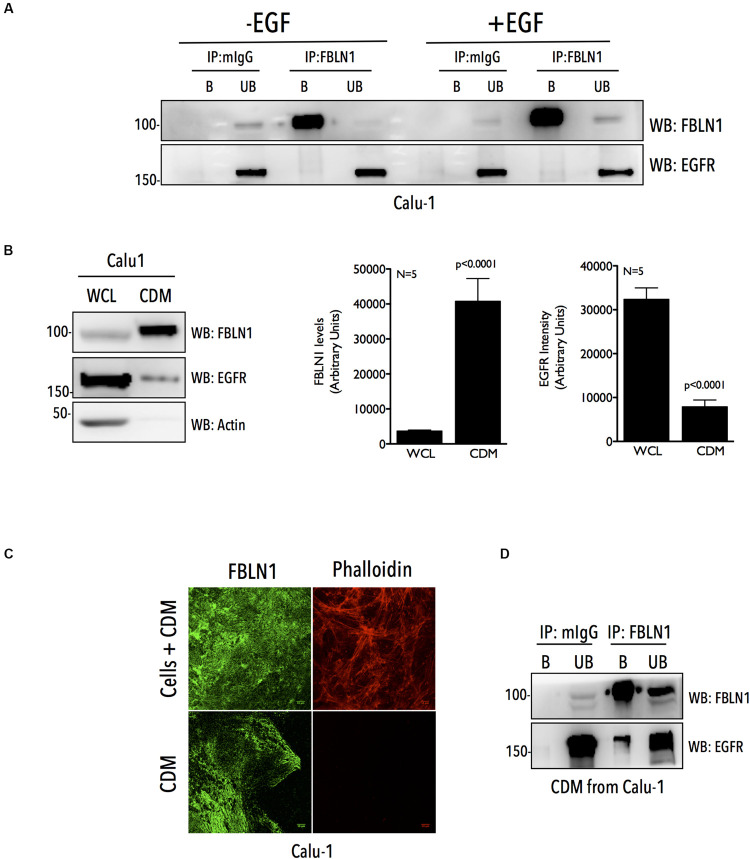
Cell derived matrix Fibulin-1 co-immunoprecipitates EGFR in Calu-1 cells. **(A)** Calu-1 cells serum starved for 12 h were stimulated with EGF (100 ng/ml) for 5 min and endogenous Fibulin-1 (IP: FBLN1) was immunoprecipitated and compared to mouse IgG (IP: mIgG). Immunoprecipitation of Fibulin-1 (WB: FBLN1) and co-precipitation of EGFR (WB: EGFR) was tested by western blot. The immunoprecipitated (bound) protein eluted and un-bound fractions (B vs. UB) were also compared by western blot. The results are representative of three independent experiments. **(B)** 10 μg of whole cell lysate (WCL) and cell derived matrix (CDM) from Calu-1 cells were probed for Fibulin-1 (WB: FBLN1), EGFR (WB: EGFR), and Actin (WB: Actin) by western blot. Bar graphs represent mean ± SE of Fibulin-1 and EGFR band intensities in CDM and WCL from five independent experiments. Statistical analysis of the data was done using the student’s *t* test and *p* values are as shown. **(C)** Calu-1 cells and CDM made from these cells were fixed and immunostained to detect FBLN1 and Actin (phalloidin alexa-594). Representative confocal images for each are shown. Scale bar represents 10 μm. Data is representative of three independent experiments with similar results. **(D)** Endogenous Fibulin-1 from CDM of Calu-1 cells was immunoprecipitated (IP: FBLN1) and compared to mouse IgG (IP: mIgG). Immunoprecipitation of Fibulin-1 (WB: FBLN1) and co-precipitation of EGFR (WB: EGFR) was tested by western blot. The immunoprecipitated (bound) protein eluted and un-bound fractions (B vs. UB) were also compared by western blot. The results are representative of three independent experiments.

We further tested if this association is detectable for endogenous FBLN1C/1D and EGFR in Calu-1 cells, where our studies show them to be functionally related (Figures 1G–J). Immunoprecipitation of endogenous FBLN1 using anti-FBLN1 antibody from WCL, failed to detect any association with EGFR (Figure 3A and Supplementary Figure 3A). This could reflect the fact that only a small fraction of the endogenous proteins bind each other or their association could be spatially or temporally regulated making it challenging to detect in whole cell lysate IP studies. FBLN1 is likely to be enriched in the matrix (Krasny et al., 2018), which could support its association and regulation of EGFR. To test this, we used a detergent free decellularization protocol to isolate cell-derived matrix (CDM). We compared equal protein from CDM and whole cell lysate of Calu-1 cells and confirmed a 11 fold enrichment of FBLN1 in the CDM (Figure 3B). Immunostaining of this CDM, further confirmed the presence of FBLN1 (Figure 3C), with decellularization confirmed by the absence of any detectable staining with phalloidin (Figure 3C and Supplementary Figure 3B). EGFR levels while much less were detected in these CDM preparations (Figure 3B) allowing us to test for its association with FBLN1. Immunoprecipitation of FBLN1 from the CDM does detect the co-immunoprecipitation of matrix associated EGFR (Figure 3D), unlike in WCL (Figure 3A). We also evaluated the association of matrix FBLN1 and EGFR in NSCLC A549 cells. A549 cells are known deposit less matrix as compared to Calu-1 cells (Andriani et al., 2016; Peixoto et al., 2019). FBLN1 levels in A549 CDMs were hence not as enriched (Supplementary Figure 3C) when compared to Calu-1 CDMs (Figure 3B). Immunoprecipitation of FBLN1 from WCL (Supplementary Figure 3D) and CDM (Supplementary Figure 3E) of A549 cells could detect FBLN1 association with EGFR only in the CDM (Supplementary Figure 3E), similar to that of Calu-1 cells. Together these findings confirm the association of endogenous FBLN1 with EGFR to be prominent in the CDM.

### Matrix Derived Fibulin-1 Regulates EGFR Activation and Localization to Control Cell Adhesion and Spreading

**FIGURE 4:**
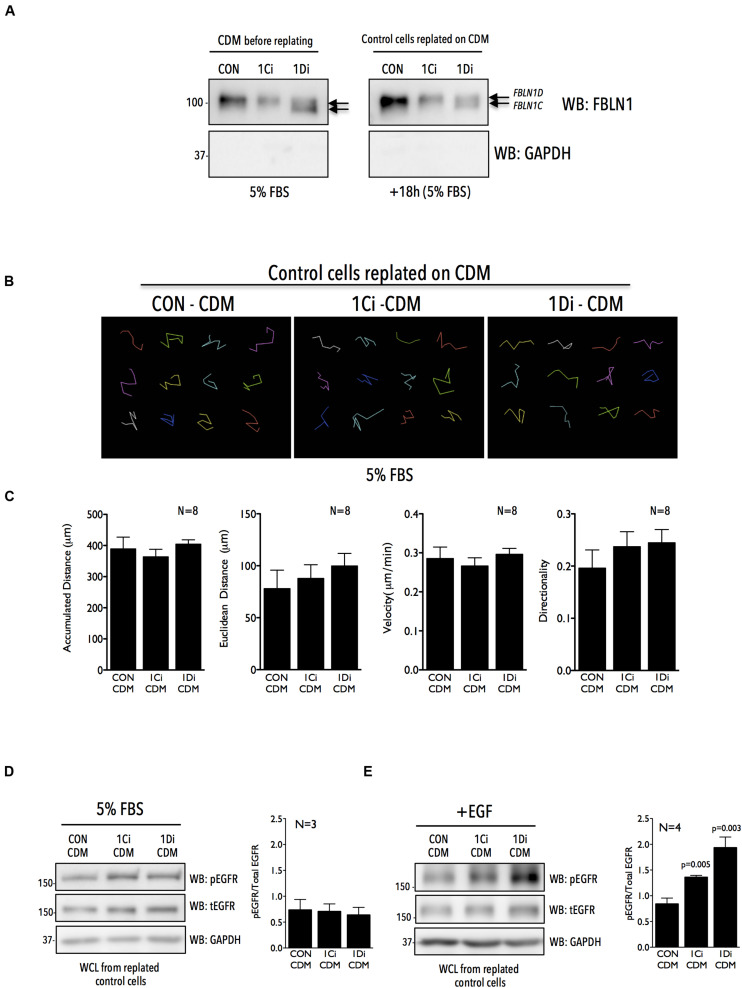
Matrix Fibulin-1 regulates EGF dependent EGFR activation in Calu-1 cells. **(A)** Endogenous Fibulin-1 (WB: FBLN1) and GAPDH (WB: GAPDH) were detected by western blot in cell derived matrix (CDM before replating) made from control (CON), FBLN1C (1Ci) and FBLN1D (1Di) knockdown Calu-1 cells, grown with serum (5% FBS) for 72 h. Calu-1 cells were replated on this CDM and grown for 18 h with serum [+18 h (5% FBS)] and levels of Fibulin-1 (WB: FBLN1) and GAPDH (WB: GAPDH) detected by western blot in cell derived matrix (CDM after replating) and compared to those seen in CDM before replating. FBLN1C and FBLN1D isoforms detected in blots of knockdown lysates are marked by arrows. Blot is best representative of three independent experiments that gave similar results. **(B)** Representative individual migration tracks of Calu-1 cells adherent in the presence of serum growth factors (5% FBS) on CDM (made as detailed above) from control (CON-CDM), FBLN1C (1Ci-CDM), and FBLN1D (1Di-CDM) knockdown Calu-1 cells. **(C)** Accumulated distance, euclidean distance, velocity and directionality of 100 migrating cells (per experiment) and represented in the bar graphs as mean ± SE from eight independent experiments. *p* values were calculated using one-way ANOVA and Tukey’s *post hoc* test and represented if found to be significant. **(D,E)** Western blot detection of EGFR phosphorylated on tyrosine 1173 (WB: pEGFR), total EGFR (WB: tEGFR) and GAPDH (WB: GAPDH) in Calu-1 plated on CDM (made as detailed above) from control (CON-CDM), FBLN1C (1Ci-CDM) and FBLN1D (1Di-CDM) knockdown cells **(D)** in the presence of serum growth factors (5% FBS) **(E)** on stimulation with EGF (100 ng/ml) for 5 min (+EGF) in serum deprived Calu-1 cells. Bar graphs represents mean ± SE of pEGFR to total EGFR ratio from 3 or 4 independent experiments as indicated. Statistical analysis of the data was done using the students *t*-test and *p* values are as shown.

The ECM has been known to act as a reservoir for growth factors and growth factor receptors to modulate cellular behavior (Grahovac and Wells, 2014; Zhu and Clark, 2014; Hastings et al., 2019). To determine if matrix derived FBLN1, seen to bind EGFR (Figure 3D), can regulate its activation and function in Calu-1 cells, we isolated CDM from control, FBLN1C and FBLN1D knockdown cells and tested their role in regulating EGFR activation and migration in replated Calu-1 cells. siRNA mediated knockdown of FBNL1C and FBLN1D in WCL of Calu-1 cells is specific (Figure 1F) which is reflected in their CDMs (Figures 1F, 4A and Supplementary Figure 4A). Western blot detection of FBLN1 in knockdown CDMs from Calu-1 cells grown with serum showed FBLN1C/1D isoforms to retain their differential mobility as reported earlier (Figures 4A, 1F). The protein content of CDMs made by similar number of control vs. knockdown cells was largely comparable (Supplementary Figures 4B,C). CDMs thus derived from control and knockdown Calu-1 cells were used to replate untreated Calu-1 cells and their function compared (Figure 4A). Re-plating of Calu-1 cells on knockdown CDMs for 18 h did not visibly affect FBLN1 levels (Figure 4A right panel). We hence tested the migration of individual Calu-1 cells replated on CDMs in the presence of serum growth factors. This revealed no significant difference in the distance, velocity and directionality of Calu-1 cells (Figures 4B,C) on control vs. FBLN1C vs. FBLN1D knockdown CDMs (Supplementary Figure 4D). We hence tested the EGFR activation status in Calu-1 cells replated on CDMs for 18 h in the presence of serum and noted no change in the EGFR activation on knockdown CDMs, relative to control (Figure 4D and Supplementary Figure 4E).

**FIGURE 5:**
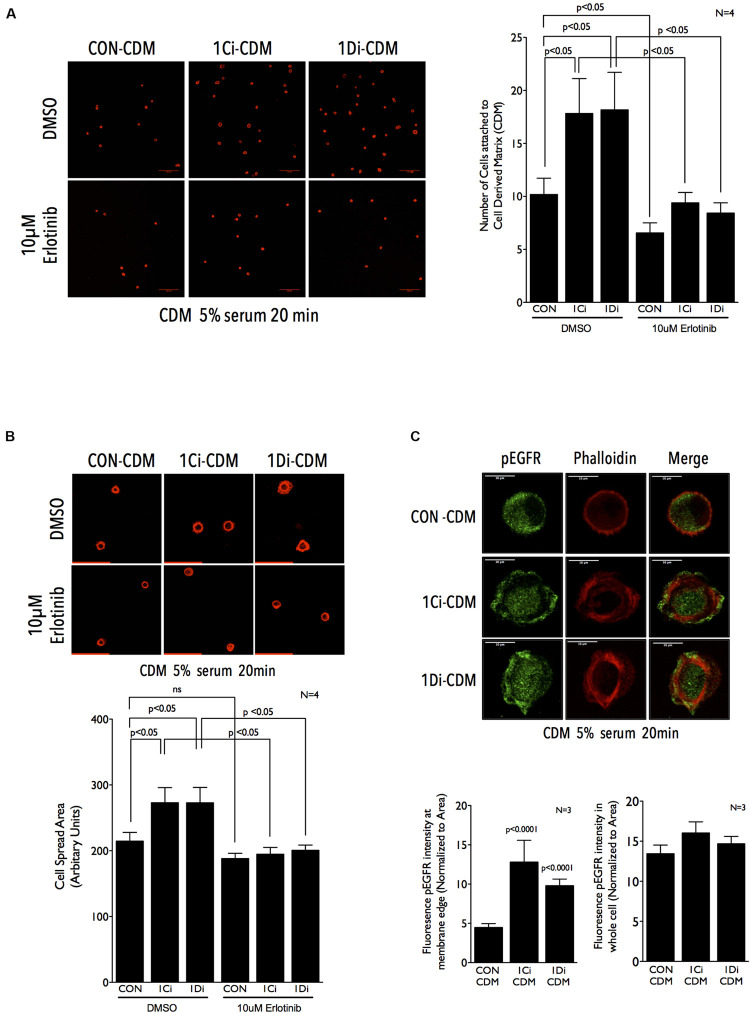
Matrix Fibulin-1 regulates EGFR-dependent adhesion and spreading in re-adherent Calu-1 cells. **(A,B)** Representative images of Calu-1 cells re-adherent for 20 min in the presence of serum growth factors (5% FBS) with DMSO or 10 μm Erlotinib on CDM from control (CON-CDM), FBLN1C (1Ci-CDM) and FBLN1D (1Di-CDM) knockdown Calu-1 cells. Bar graph represent mean ± SE of **(A)** number of cells attached **(B)** cell spread area, from 10 or more frames each in four independent experiments. Statistical analysis of the data was done using two-way ANOVA and *p* values are as shown. **(C)** Representative images of Calu-1 cells replated for 20 min in the presence of on CDM from control (CON-CDM), FBLN1C (1Ci-CDM) and FBLN1D (1Di-CDM) knockdown Calu-1 cells shows the localization of EGFR phosphorylated on tyrosine1173 (pEGFR) and actin cytoskeleton (phalloidin), with merged images. Scale bar represents 10 μm. The intensity of pEGFR in region of 0.5 μm from the cell edge was measured and normalized to its area (left graph). The same was done for the entire cell as well (right graph) and compared between control (CON), FBLN1C (1Ci-CDM) and FBLN1D (1Di-CDM) knockdown Calu-1 cells. The bar graph represents mean ± SE of these from three independent experiments. Statistical analysis of the data was done using two-way ANOVA and *p* values are as shown.

Since this reflected a more sustained growth factor mediated activation of EGFR on knockdown CDMs, we asked if a more rapid and robust activation by externally added EGF (5 min stimulation) can change the cellular response. Indeed, on EGF stimulation EGFR activation was significantly better in cells plated on FBLN1C (∼1.5 fold) and FBLN1D knockdown (∼2.0 fold) CDMs, relative to control CDM (Figure 4E and Supplementary Figure 4F). This is comparable to results seen on EGF stimulation of FBLN1 KD Calu-1 cells (Figure 1H), suggesting matrix associated Fibulin-1 to be a major mediator of this regulatory crosstalk. Such a rapid activation of EGFR is reported on re-adhesion of cells to ECM promoting cell binding and spreading (Normanno et al., 2006; Guo et al., 2015). We hence tested the possible impact Fibulin-1 in the CDM could have in mediating re-adherent Calu-1 cell adhesion and spreading. Cells were detached and replated on control vs. FBLN1 knockdown CDMs for 20 min to trigger integrin signaling and EGFR activation to drive adhesion and cell spreading (Su and Besner, 2014; Lopez-Luque et al., 2019). Cells replated on FBLN1C knockdown and FBLN1D knockdown derived CDMs attached and spread significantly better than control CDMs, which was significantly reduced by Erlotinib mediated inhibition of EGFR (Figures 5A,B and Supplementary Figure 5A). We further looked at the localization of activated EGFR (pEGFR) in spreading cells and found it to be enriched at membrane ruffles in cells replated on FBLN1C knockdown CDM and FBLN1D knockdown CDM, relative to control CDM (Figure 5C and Supplementary Figure 5B). Since EGFR has been known to be transactivated in a ligand independent manner via integrin binding to Fibronectin (Guo et al., 2015), we tested the levels of Fibronectin (FN) in Calu-1 cells upon FBLN1C and FBLN1D knockdown and did not see any differences (Supplementary Figure 5C). Taken together these studies identify a role for matrix bound FBLN1 to spatially regulate EGFR activation in Calu-1 cells to regulate cell function.

## Discussion

As part of the tumor microenvironment the ECM not only provides structural support but through the biophysical and biochemical cues creates a dynamic microenvironment that helps cancer cells evade growth suppression, acquire resistance to apoptosis and initiate metastasis (Lu et al., 2012; Pickup et al., 2014; Walker et al., 2018). ECM molecules like Fibulin-3, Tenascin-C and Emilin-2 have been known to associate with growth factor receptors like EGFR to regulate its activation and function during cancer progression (Iyer et al., 2008; Grahovac and Wells, 2014; Kim et al., 2014; Paulitti et al., 2018). Fibulins as part of the ECM are seen to interact with various basement membrane proteins and elastic fibers (Argraves et al., 2003; Chu and Tsuda, 2004). FBLN2, FBLN3, and FBLN5 have been shown to regulate Notch (Baird et al., 2013), Insulin Growth Factor receptor (IGF1R) (Kim et al., 2014), EGFR signaling pathways, respectively, in glioma (Hu et al., 2009), lung (Yue et al., 2009; Kim et al., 2014; Wang et al., 2017), and pancreatic cancers (Camaj et al., 2009). Both FBLN1, FBLN3 are highly expressed in normal adult lung and downregulated in lung cancer cells (NSCLC) (Yue et al., 2009; Kim et al., 2014; Cui et al., 2015). FBLN3 is also shown to directly bind EGFR (Camaj et al., 2009) and affects its activation in NSCLC, though its effect on EGFR mediated tumor progression is marginal (Kim et al., 2014). TCGA and Oncomine data both show FBLN1 (and FBLN3–data not shown) to be downregulated in NSCLC, with significant overexpression of EGFR (Figures 1A–D; Okayama et al., 2012; Selamat et al., 2012; Campbell et al., 2016). This study in identifying a role for matrix FBLN1 in regulating EGFR activation in NSCLC Calu-1 cells further adds to our understanding of this regulatory crosstalk in lung cancers. Our results show that FBLN1 suppresses EGFR activation, and that loss of FBLN1C/FBLN1D (as seen in lung cancers) can promote EGFR dependent cellular function.

Structurally, Fibulin-1 comprises of several EGF like modules that could be important for its association with EGFR, as seen for other matrix proteins (Argraves et al., 1990; Timpl et al., 2003; Grahovac and Wells, 2014). FBLN1 has also been shown to interact with other EGFR interacting proteins like Versican, Fibronectin and HB-EGF, which could indirectly mediate the association of FBLN1 with EGFR. It would also be interesting to determine whether FBLN1C and FBLN1D also bind EGFR ligands (i.e., EGF and HB-EGF) differentially and the impact that has on this regulation. Such a regulation could also contribute to the differential effects seen in wound healing assays, possibly occurring as a result of increased HB-EGF shedding which could have an effect on EGFR activation and cell migration (Brooke et al., 2002; Yin et al., 2007) as seen in Figure 1H. Both the FBLN1 isoforms, FBLN1C and FBLN1D bind EGFR (Figures 2A–D), and the enrichment of FBLN1 in CDM allows the detection of its association with EGFR (Figure 3D). Similar to other ECM proteins that bind EGFR, FBLN1 mediated tethering of EGFR to the ECM can limit its availability for ligand binding on the cell surface via steric hindrance (Santra et al., 2002; Camaj et al., 2009), this by affecting its clustering and dimerization can also regulate its activation (Ferguson et al., 2003; Tsai and Nussinov, 2019). Further, the stability, internalization and degradation of EGFR could also be affected by matrix FBLN1 to regulate its cellular function. EGFR activation does not affect its association with FBLN1C or 1D, which is distinctly different from FBLN3 (Camaj et al., 2009; Hu et al., 2014). This could further drive the impact FBLN1 downregulation has on EGFR dependent lung cancer cells (NSCLC) (Yue et al., 2009; Kim et al., 2014; Cui et al., 2015).

Fibulin-1 isoforms in Calu-1 cells show a distinct difference in their mobility on SDS PAGE, reflecting possible changes in their post-translational modification (Argraves et al., 1990; Aspberg et al., 1999), which is retained in the FBLN1 secreted in the conditioned culture medium and matrix bound FBLN1 (Figure 1F). This is further evident in isoform specific knockdowns of FBLN1C/1D (Figure 1F) and their overexpression (Figure 1I). Fibulin-1 isoforms differ marginally in their relative effects on EGFR activation, suggesting their regulation of its activation and function could possibly be context dependent. One of the regulating factors could be the nature of EGF stimulation. In response to sustained growth factor mediated EGF stimulation (5% FBS) FBLN1C and FBLN1D comparably affect EGFR activation in Calu-1 cells (Figure 1G). Rapid and robust activation of EGFR by stimulation with EGF (100 ng/ml for 5 min) revealed a more pronounced and differential regulation by FBLN1 isoforms, with FBLN1D seemingly a more potent regulator (Figures 1H, 4E). This effect is seen in Calu-1 cells lacking FBLN1D (Figure 1H) and Calu-1 cells plated on FBLN1D knockdown CDMs (Figure 4E).

The differential stimulation of EGFR on FBLN1 mediated cell function is further evident in the effect FBLN1C, FBLN1D knockdown has on migration in the wound healing versus single cell migration of Calu-1 cells re-plated on knockdown CDMs. With FBLN1 enriched in CDM we expect the behavior of Calu-1 cells in both these conditions to be comparable. However, in wound healing assays, loss of FBLN1C and 1D promotes Calu-1 cell migration, while this effect is not seen in single cell migration when control Calu-1 cells are re-plated on FBLN1C and 1D knockdown CDMs in the presence of 5% FBS. Calu-1 cells plated on FBLN1 knockdown CDMs in the presence of 5% FBS show no differential EGFR activation (Figure 4D), unless cells are rapidly stimulated with externally added EGF (Figure 4E). This suggests that the nature of EGFR stimulation (Serum vs. EGF) could affect the impact FBLN1 has on cellular function. In wound healing assays, cellular damage by the scratch releases ATP, which stimulates the shedding of HB-EGF mediating the transactivation of EGFR (Yin et al., 2007) could drive the differential migration of FBLN1 knockdown cells. Studies have shown FBLN1C binds HB-EGF (Brooke et al., 2002), which is a prominently overexpressed ligand for EGFR in NSCLC (Hsieh et al., 2017; Yotsumoto et al., 2017). This could in part contribute to the differential effect FBLN1C has relative to FBLN1D in EGFR dependent migration and wound closure.

Epidermal growth factor receptor has been known to play a major role in mediating cell adhesion (Andl et al., 2003; Abu-Ali et al., 2008; Ungewiss et al., 2018) and cell spreading (Marcoux and Vuori, 2003; Abu-Ali et al., 2008; Balanis et al., 2011; Morello et al., 2011). EGFR mediated activation of PKC is known to regulate cell matrix adhesion in breast and brain cancers (Sun et al., 2005; Micallef et al., 2009). Integrin mediated adhesion is seen to regulate EGFR activation by recruiting Vav2 (GEF for Rac) via PI3 kinase to promote cell spreading (Thalappilly et al., 2010). EGFR also binds FAK via SRC-3Δ4 adaptor molecule and phosphorylates it at Y925 to promote cancer cell migration and invasion (Long et al., 2010). Inhibition of EGFR disrupts cell spreading on fibronectin, with reduced membrane ruffles (Thalappilly et al., 2010). CDM mediated rapid stimulation of EGFR in Calu-1 cells regulates cell adhesion and its localization at lamellopodial ruffles and cell spreading (Figure 5). Matrix Fibulin-1 levels by affecting ECM composition and architecture could influence the integrin-EGFR crosstalk in these cells. Although, no differences in the levels of Fibronectin are seen upon FBLN1 knockdown in Calu-1 cells (Supplementary Figure 5C), further studies are required to evaluate the effects loss of Fibulin-1 has on Fibronectin polymerization and its incorporation into the ECM. With the known role EGFR activation and localization to the mitochondria has in lung cancer cells (Che et al., 2015), evaluating the impact matrix Fibulin-1 could have in mediating mitochondrial function would be of much interest.

Cancer cell secreted matrix proteins and growth factors are known to support tumor invasion (Walker et al., 2018; Hoshiba, 2019). Studies have shown CDM from cancer cells drive cell adhesion, migration, invasion, angiogenesis and chemo resistance (Senthebane et al., 2017; Hoshiba, 2019; Nallanthighal et al., 2019). Changes in the composition of the cancer CDM can hence affect neighboring cancer and normal cells influencing tumor organization and progression (Attieh et al., 2017; Erdogan et al., 2017; Paolillo and Schinelli, 2019). Non-small-cell lung cancers (NSCLCs) have diverse pathological features, that are a result of genetic and cellular heterogeneity (Chen et al., 2014). Several components including origin of cells, genetic alterations and microenvironmental factors all contribute to the lineage identity of lung tumors (Fong et al., 2003; Sharma et al., 2007; Chen et al., 2014; Malik and Raina, 2015). Cell-derived matrix from FBLN1C/FBLN1D knockdown Calu-1 cells regulating EGFR and its function (cell adhesion and spreading) mimics the loss of FBLN1 phenotype in Calu-1 cells. The possible overlap and differences in the role FBLN1C/1D on EGFR function could extend the impact tumor microenvironments could have in regulating cell signaling and behavior in heterogenous lung tumors.

## Materials and Methods

### Cell Culture

Calu-1 cells (ECACC), A549 (ECACC), and HEK293T (Obtained from Dr. Aurnab Ghose, IISER Pune) cells were cultured in Dulbecco’s Modified Eagles Medium (DMEM) High, supplemented with 5% v/v FBS (Invitrogen), and 1% v/v Penicillin/Streptomycin (Invitrogen). Media was changed every 3 days and cells were passaged at 70–80% confluency with Trypsin (Invitrogen). Cell lines were grown at 37°C under 5% CO_2_. All cell lines were routinely tested for mycoplasma contamination.

### Plasmids and siRNAs

Untagged FBLN1C and FBLN1D constructs were obtained from Dr. Marion Cooley (Augusta University, Augusta, GA, United States). GFP tagged EGFR construct was bought from Addgene. Sequences of all the constructs were verified before use. FBLN1C and FBLN1D siRNAs were designed using Dharmacon Design Center and synthesized in duplex form from Sigma with [dT] overhangs under standard desalting conditions. The siRNA’s were reconstituted with nuclease free water and stored at −20°C until further use.

### Transfections and Knockdowns

Cells were transfected using Polyethanolamine (PEI) (Sigma) according to the manufacturer’s protocol. Transfections were done in 6-well plates or 10-cm dishes with complete medium using 2 μg or 10 μg DNA, respectively, for 48 h (for all constructs used). At 48 h after transfection, cells were serum deprived for 12 h in low-serum DMEM (containing 0.2% FBS) and then used for immunoprecipitation (IP) experiments. Calu-1 and A549 cells transfected with FBLN1C or FBLN1D were subjected to cell lysis at the end of 48 h post transfection.

siRNA mediated knockdown was performed using RNAiMAX (Invitrogen). Cells were plated for 6 h at a density of 2.4 × 10^5^ per well in a six well plate followed by first shot of FBLN1C (5 picomoles) and FBLN1D siRNA knockdown (5 picomoles). Media was changed 24 h post knockdown along with a second shot of knockdown. Media was changed 24 h post second shot knockdown and the cells were trypsinized, counted and replated for assays as described below.

### Preparation of Conditioned Culture Media (CCM)

Control, FBLN1C and FBLN1D knockdown Calu-1 cells (2.5 × 10^5^ cells/well) were grown for 72 h (upon replating) in six well plates without media change. At then end of 72 h, 500 ul of the CCM from the CON, FBLN1C and FBLN1D knockdown Calu-1 cells was collected. CCM was then centrifuged at 1000 rpm for 5 min at room temperature (RT) to remove any cell debris. 400 ul of this supernatant CCM was collected and 100 ul of 5X laemmli added, boiled at 95°C, cooled and used for SDS PAGE.

### RNA Extraction and qRT-PCR

RNA was isolated using TRIzol (Invitrogen) and cDNA was prepared from total RNA using iScript cDNA synthesis kit (Bio-Rad) according to the manufacturer’s specifications. Undiluted cDNA was used in 5 μl quantitative PCR (qRT-PCR) reaction with SYBR FAST qPCR master mix (Kapa Biosystems) in a Bio-Rad CFX96 Real Time System using the following human primer sequences. FBLN1C 5′ caactgctccatcaacgaga 3′ (Forward), 5′ attctcagaggcagcttgga 3′ (Reverse), FBLN1D 5′ cgagtgccctgagaactacc 3′ (Forward), 5′ gagatgacggtgtgggagat 3′ (Reverse), EGFR 5′ gatacccaggaccaagccac 3′ (Forward), 5′ ggaatgcaacttccaaaatgtg 3′ (Reverse), Actin 5′ ctcctgagcgcaagtactcc 3′ (Forward), 5′ ccggactcgtcatactcctg 3′ (Reverse). All samples were amplified in triplicates. Fold change in gene expression relative to control was calculated using the delta delta Ct.

### Antibodies and Reagents

The following antibodies were used for western blotting: mouse anti-FBLN1 (Santa Cruz Biotechnology SC25281) at 1:1000 dilution, mouse anti-Fibronectin (DSHBS1-1634) at 1:250 dilution, rabbit anti-EGFR (Cell Signaling 2232S) at 1: 1000 dilution, rabbit anti-phospho-EGFR Y1173 (R&D systems AF1095) at 1:1000 dilution, mouse anti- βActin (Abcam Clone ACTN05 (C4) Ab3280) at 1:2000 dilution and rabbit anti-GAPDH (G9545 Sigma Aldrich) at 1:5000 dilution. Horseradish Peroxidase conjugated secondary antibodies (Anti-Mouse and Anti-Rabbit) were used at a dilution of 1:10,000 and were purchased from Jackson Immuno Research.

Opti-MEM was purchased from Invitrogen (cat. no. 22600-050). Ammonium Hydroxide (NH_4_OH, 05002-1L), DMSO (D2438) and EGF was purchased from Sigma (E9644). Erlotinib Hydrochloride was purchased from Santa Cruz Biotechnology (CAS- 183319-69-9). Protein A Sepharose beads (GE 17-0780-01) were purchased from Sigma Aldrich. Mouse IgG (Millipore 12-371) and Rabbit IgG (Millipore 12-370) were also purchased from Sigma Aldrich.

Alexa Fluor 488/594 conjugated to Phalloidin (Invitrogen, cat. no. A12379 and A12381) was used at a dilution of 1:400. Secondary antibodies conjugated to Goat anti-mouse IgG Alexa Fluor 488, Goat anti-Rabbit IgG Alexa Fluor 488 were used at a dilution of 1:500 and was purchased from Invitrogen Molecular Probes (cat. no. A-11029 and A-11008). Fluoromount-G (cat. no. 0100-01) was purchased from Southern Biotech.

### Immunofluorescence Staining for Fibulin-1 and Phosphorylated EGFR (Y1173)

Calu-1 Cells or CDM on coverslips were fixed with 3.5% paraformaldehyde (PFA) in phosphate buffered saline (PBS) for 15 min at RT. Cells were permeabilized with PBS containing 5% bovine serum albumin (BSA) and 0.05% Triton X-100 for 15 min at RT. Blocking was done with 5% BSA in PBS for 60 min at RT followed by incubation with anti-Fibulin-1 (1:100, 90 min incubation at RT) or anti-phosphorylated EGFR (Y1173) antibody (1:100, 180 min incubation at RT) diluted in 1% BSA in PBS. The coverslips were then washed three times for 5 min each with 0.1% BSA in PBS. Coverslips were then incubated with anti-mouse Alexa Flour 488 (1:500) and Alexa Flour 594 Phalloidin (1:500) in 1% BSA in PBS for 1 h at RT. The coverslips were then washed three times for 5 min each with 0.1% BSA in PBS and mounted using Fluoromount-G and left to dry for 24 h followed by imaging using a confocal microscope.

### Preparation of Cell Derived Matrix (CDM)

#### Control CDM for Western Blotting and Immunoprecipitation Experiments

Calu-1 cells were plated at a density of 4 × 10^5^ cells in a 100 mm dish and cultured for 5 days. Cells reached 100% confluency at the end of day 5. On day 6, media was aspirated followed by a gentle PBS wash. The cells were then treated with 20 mM NH_4_OH (made with Distilled Water) for 4 min followed by three washes with distilled water (2 min each). Decellularization was confirmed when the cells were no longer visible when visualized under the microscope. CDM left back on the plate was lysed and the lysates were protein estimated and subjected to Immunoprecipitation (IP) and western blotting.

#### FBLN1C, FBLN1D Knockdown CDM for Cell Adhesion, Cell Spreading and pEGFR Immunostaining

Calu-1 cells which were subjected to FBLN1C, FBLN1D knockdown were used to make CDM on coverslips. Briefly, 24 h post second shot knockdown (KD) as described in the section “Transfections and Knockdowns,” cells were trypsinized and replated on coverslips in a six well plate at a density of 2.5 × 10^5^ cells per well and grown for 72 h. CDM was made from FBLN1C and FBLN1D knockdown cells by decellularization using 20 mM NH_4_OH (made with sterile distilled water) for 4 min followed by two washes (2 min each) of sterile distilled water. The coverslips were then washed with sterile PBS and blocked for 30 min with 5% serum containing medium (with and without Erlotinib) to be used for further experiments.

#### Cell Adhesion and Spreading Assay

Calu-1 cells were plated at a density of 4 × 10^5^ cells in a 60 mm dish and cultured for 24 h. Cells treated with DMSO or Erlotinib (10 μM) for 6 h were replated at a density of 4 × 10^4^/well in a six well plate on control CDM, FBLN1C knockdown CDM and FBLN1D knockdown CDM for 20 min. Erlotinib was maintained in the media during all steps of processing. Cells were then fixed with 3.5% PFA at RT followed by Phalloidin staining. Confocal images of cells attached to CDM were analyzed using Image J software (NIH). Number of cells attached to the CDM in each frame was counted using cell count tool in Image J. For calculating cell spread area, thresholding was done to select the entire cell and the tracing tool was used to select the edge of the cell. Wand tool was used to measure the area of the cell within the mapped edge. 10 images per group were used to calculate number of cells attached to CDM. For calculating cell-spread area, at least 75 cells per group in each experiment were used for analysis.

### Co-immunoprecipitation Experiments

#### HEK 293T Cells

HEK293T cells transfected with 5 μg of FBLN1C or 5 μg of FBLN1D and 5 μg of EGFR-GFP with PEI for 48 h. For experiments with and without EGF stimulation, cells were serum starved with 0.2% serum containing media for 12 h followed by a 5 min treatment with 100 ng/ml of EGF. Cells were lysed with 20 mM HEPES 7.8, 120 mM NaCl, 10% Glycerol, 1% NP40, 5 mM EDTA, 1 mM MgCl2, 5 mM NaF, 4 mM Vanadate, 1 mM PMSF and 1X PIC) for 30 min on ice followed by centrifugation at 12,000 rpm for 30 min at 4°C. Supernatant was collected and the lysate was protein estimated using BCA kit. Lysates were precleared with 20 μl of protein A sepharose beads in end to end rotor for 1 h at 4°C. 500 μg of lysate used for each IP reaction was incubated for 1 h at 4°C on end to end rotor with anti-EGFR (2 μl) or FBLN1 antibodies (1 μg) and their respective Ms IgG/Rb IgG (1 μg) controls. Lysates with the antibodies were incubated with 40 μl of Protein A sepharose bead slurry for 2 h at 4°C on end to end rotor. Antibody coupled beads were washed thrice with lysis buffer to remove unbound fractions. Beads containing the immunecomplexes were transferred to a different tube after third wash and eluted using 40 μl of 1X Laemmli buffer and boiled at 95°C for 10 min on thermomixer (500 rpm). Bound and unbound fractions subjected to SDS-PAGE electrophoresis were probed with anti-FBLN1 and anti-EGFR antibodies and blots were developed using the LAS4000 detection system (Fujifilm-GE).

#### Calu-1 Cells

Calu-1 cells were plated at a density of 4 × 10^5^ cells in a 100 mm dish and cultured for 5 days. Cells reached 100% confluency at the end of day 5. At the end of day 5 cells were serum starved with 0.2% serum containing media for 12 h followed by a 5 min treatment with 100 ng/ml of EGF. Cell lysates were prepared and subjected to IP using anti-FBLN1 antibody as stated above.

#### Calu-1 and A549 CDM

Calu-1 cells were plated at a density of 4 × 10^5^ cells in a 100 mm dish and cultured for 5 days. Cells reached 100% confluency at the end of day 5. On day 6, media was aspirated followed by a gentle PBS wash. The cells were then treated with 20 mM Ammonium Hydroxide (made with Distilled Water) for 4 min followed by three washes with distilled water (2 min each). CDM was lysed as mentioned above and the protein estimation was done using MicroBCA kit. CDM samples were diluted using dilution buffer (20 mM HEPES 7.8, 120 mM NaCl, 1% Glycerol, 1% NP40, 5 mM EDTA, and 1 mM MgCl_2_) to make it compatible with the MicroBCA kit. CDM was precleared with 20 μl of protein A sepharose beads in end to end rotor for 1 h at 4°C. 100 μg of lysate used for each IP reaction was incubated for 1 h at 4°C on end to end rotor with anti-FBLN1 antibody (1 μg) or Ms IgG (1 μg) controls. CDM containing the immunecomplexes was then incubated with 40 ul of Protein A sepharose bead slurry for 3 h at 4°C on end to end rotor. Antibody coupled beads were washed thrice with lysis buffer to remove unbound fractions. Beads containing the immunecomplexes were transferred to a different tube after third wash and eluted using 40 ul of 1X Laemmli buffer and boiled at 95°C for 10 min on thermomixer (500 rpm). Bound and unbound fractions subjected to SDS-PAGE electrophoresis were probed with anti-FBLN1 and anti-EGFR antibodies and blots were developed using the LAS4000 detection system (Fujifilm-GE).

### Wound Healing Migration Assay

Wound healing assay was used to determine the collective cell migration of Calu-1 cells upon FBLN1C and FBLN1D knockdown. FBLN1C/FBLN1D knockdown in Calu-1 cells was performed as described above in the “Transfections and Knockdowns.” Twenty fours post second shot knockdown, cells were plated at 2 × 10^5^ cells/well in a 24 well plate and cultured overnight. Cells were treated with DMSO or Erlotinib (10 μM) for 6 h in 5% serum containing media. Cells were then treated with Mitomycin C (10 μg/ml) for 1 h to inhibit cell proliferation and scratch was performed with a 10 μl sterile pipette tip. Cells were washed with PBS to remove cell wounded cells and debris. Fresh media was added to the cells and 5 images at different locations in the scratch were taken per group at 0 h, 12 h, 24 h, and 36 h, respectively. Percentage closed wound area was calculated using T-Scratch software.

### Single Cell Migration Assay on CDM

Upon second shot knockdown, cells were plated at 5 × 10^4^ cells/well in a 24 well plate and cultured for 72 h. CDM from FBLN1C, FBLN1D knockdown cells were prepared as described in the section “FBLN1C, FBLN1D Knockdown CDM for Cell Adhesion, Cell Spreading and pEGFR Immunostaining.” Untreated Calu-1 cells were plated at 2 × 10^4^ cells/well in a 24 well plate and allowed to attach for 3 h. Images were taken every 90 min for 12 h. Cells were manually tracked using MTrack plugin in Image J software (NIH). Distance, Velocity and Persistence was calculated using Chemotaxis and Migration tool in Image J software.

### Quantitation of Fluorescent Phosphorylated EGFR in Cell Edge and Whole Cells

Confocal cross section images were shot at the plane the cells are most spread and the used for this analysis. To calculate enrichment of active phosphorylated EGFR (pEGFR) in Calu-1 cell edges cells replated on cell derived matrices from control, FBLN1C and FBLN1D knockdown cells were imaged using a laser confocal. The cell spread area (Area 1) was defined by thresholding of the Phalloidin stained cell image using Image J. This was used to define a mask for the perimeter of the cell. The area that this mask measures is called Area 1. This mask was stored in the ROI (Region of Interest) manager. This ROI was then shrunk evenly by 0.5 μm using the Enlarge option (by entering a negative 0.5 μm value). The area that this shrunk mask now measures is called Area 2. pEGFR intensity in Area 1 and Area 2 were measured and intensity in Area1 minus Area2 was calculated and normalized to the area of this region. This effectively gives us the intensity of pEGFR (active EGFR)/Area in 0.5 μm of the cell edge in the actively spreading cells.

### TCGA/ONCOMINE Analysis

Analysis of FBLN1, EGFR transcript levels from TCGA datasets was done using the normalized RNA-Seq expression data. The following datasets were downloaded from the UCSC Xena browser^1^ : TCGA Lung Cancer (LUNG) (15 datasets, *n* = 1129, version 2017-09-08), TCGA LUAD (23 datasets, *n* = 576, version 2017-10-13), and TCGA LUSC (24 datasets, *n* = 553, version 2017-10-13). FBLN1 and EGFR expression in LUAD datasets was determined using the online ONCOMINE database. The following datasets that were used for this analysis were from these studies Okayama et al. (2012) Cancer Research 2012 and Selamat et al. (2012) Genome Research 2012.

### Statistical Analysis

Statistical analysis of data was done using the unpaired two-tailed Student’s *t*-test for analyzing two groups and two tailed single sample *t* test was used for datasets normalized to respective controls. In experiments with more than three groups, two-way ANOVA was used to calculate *p* values. All analysis was done using GraphPad PRISM software.

## Data Availability Statement

All datasets generated for this study are included in the article/Supplementary Material.

## Author Contributions

KH and NB conceptualized the study based on preliminary results and wrote the manuscript. KH designed and executed all the experiments in the manuscript. OJ performed the Fibronectin experiments. SM conducted the Fibulin-1 in CDM experiments. All authors provided comments and feedback on the manuscript and approved the final draft.

## Conflict of Interest

The authors declare that the research was conducted in the absence of any commercial or financial relationships that could be construed as a potential conflict of interest.

## Footnotes

1 https://xenabrowser.net/datapages/

## Supplementary Material

FIGURE S1 **(A) Supplementary Table S1** lists custom designed siRNA sequences against FBLN1C and FBLN1D used for the knockdown experiments. **(B)** RTPCR analysis of FBLN1C (gray bar) and FBLN1D (white bar) transcript levels in siRNA-mediated knockdown of FBLN1C (FBLN1Ci) and FBLN1D (FBLN1Di) in Calu-1 cells compared to their control (CON) (black bar) (Figures 1F,G). Delta Delta Ct calculated relative to control was used to determine gene expression. Graph represents mean ± SE of relative gene expression from four independent experiments. **(C,D)** RTPCR analysis of FBLN1C (gray bar) **(B)**, FBLN1D (white bar) **(B)** and EGFR (black bar) **(C)** in siRNA-mediated knockdown of FBLN1C (FBLN1Ci) and FBLN1D (FBLN1Di) in Calu-1 cells compared to their control (CON) (black bar) (Figures 1H,I). Delta Delta Ct calculated relative to control (CON) was used to determine fold change in gene expression. Graph represents mean ± SE of relative gene expression from nine independent experiments. **(E)** Comparison of western blots for the detection of FBLN1 (WB: FBLN1) in conditioned culture medium of Control (CON), FBLN1C (Ci) and FBLN1D (Di) knockdown cells (adapted from Figure 1F) and whole cell lysates of Calu-1 cells overexpressing FBLN1C (+1C) and FBLN1D (+1D) in the presence of serum (5% FBS) (adapted from Figure 1I). Blue and red arrows mark FBLN1C and FBLN1D, respectively. Images are representative of four independent experiments. **(F)** Western blot detection of EGFR phosphorylated on tyrosine 1173 (WB: pEGFR), total EGFR (WB: tEGFR) and GAPDH (WB: GAPDH) in Calu-1 cells grown with serum growth factors (5% FBS) and treated with DMSO or 10 μM Erlotinib. Bar graphs represents mean ± SE of pEGFR to total EGFR ratio from three independent experiments. Statistical analysis of the data was done using the one sample *t*-test and *p* values are as shown. **(G)** RTPCR analysis of FBLN1C (gray bar) and FBLN1D (white bar) transcript levels in siRNA-mediated knockdown of FBLN1C (FBLN1Ci) and FBLN1D (FBLN1Di) compared to control (CON) (black bar) A549 cells. Delta Delta Ct calculated relative to control was used to determine gene expression. Graph represents mean ± SE of relative gene expression from four independent experiments. **(H)** Western blot detection of EGFR phosphorylated on tyrosine1173 (WB: pEGFR), total EGFR (WB: tEGFR) and GAPDH (WB: GAPDH) in lysates from A549 cells grown in the presence of serum growth factors (5% FBS) overexpressing untagged Fibulin-1C (+FBLN1C) and Fibulin-1D (+FBLN1D). Overexpression of FBLN1C and FBLN1D was confirmed by western blot (WB: FBLN1) and their relative positions marked by arrows. Bar graphs represents mean ± SE of pEGFR to total EGFR ratio from five independent experiments. Statistical analysis of the data was done using the students *t*-test and *p* values are as shown.

FIGURE S2 **(A,B)** Western blot detection of EGFR phosphorylated on tyrosine1173 (WB: pEGFR), total EGFR (WB: tEGFR), Fibulin-1 (WB: FBLN1), and Actin (WB: Actin) in lysates from serum deprived HEK293T cells **(A)** overexpressing EGFR-GFP and untagged FBLN1C or **(B)** EGFR-GFP and untagged FBLN1D and treated without (-EGF) or with stimulation using EGF (100 ng/ml) for 5 min (+EGF). Data is representative of three independent experiments with similar results.

FIGURE S3 **(A)** Western blot detection of EGFR phosphorylated on tyrosine1173 (WB: pEGFR), total EGFR (WB: tEGFR), Fibulin-1 (WB: FBLN1) and GAPDH (WB: GAPDH) in lysates from serum deprived Calu-1 cells without (-EGF) on with stimulation using EGF (100 ng/ml) for 5 min (+EGF). Data is representative of three independent experiments with similar results. **(B)** CDM from Calu-1 cells fixed and immunostained using Alexa 488 conjugated mouse IgG (MsIgG) and phalloidin alexa-594 (Phalloidin). Representative confocal images are representative of three independent experiments with similar results. Scale bar represents 10 μm. **(C)** 10 μg of whole cell lysate (WCL) and cell derived matrix (CDM) from A549 cells were probed for Fibulin-1 (WB: FBLN1), EGFR (WB: EGFR), and Actin (WB: Actin) by western blot. The results are representative of three independent experiments. **(D,E)** Endogenous Fibulin-1 from **(D)** WCL, Fibulin-1 from CDM **(E)** of A549 cells was immunoprecipitated (IP: FBLN1) and compared to mouse IgG (IP: mIgG). Immunoprecipitation of Fibulin-1 (WB: FBLN1) and co-precipitation of EGFR (WB: EGFR) was tested by western blot. The immunoprecipitated (bound) protein eluted and un-bound fractions (B vs. UB) were also compared by western blot. The results are representative of three independent experiments which gave similar results.

FIGURE S4 **(A)** Bar graphs represent mean ± SE of FBLN1 levels in CDM from 4 independent experiments normalized to control (CON). Statistical analysis of the data was done using the single sample *t*-test and *p* values are as shown. **(B)** RTPCR analysis of FBLN1C (gray bar) and FBLN1D (white bar) transcript levels in siRNA-mediated knockdown of FBLN1C (FBLN1Ci) and FBLN1D (FBLN1Di) in Calu-1 cells compared to their control (CON) (black bar). Delta Delta Ct calculated relative to control was used to determine gene expression. Graph represents mean ± SE of relative gene expression from four independent experiments as indicated. **(C)** Bar graphs represent mean ± SE of protein concentration in CDM quantified using BCA from four independent experiments as indicated. **(D–F)** RTPCR analysis of FBLN1C (gray bar) and FBLN1D (white bar) transcript levels in siRNA-mediated knockdown of FBLN1C (FBLN1Ci) and FBLN1D (FBLN1Di) in Calu-1 cells compared to their control (CON) (black bar) **(D)** in single cell migration (Figure 4C) **(E)** in EGFR activation studies with serum growth factors (Figure 4D) and **(F)** without serum growth factors (Figure 4E). Graph represents mean ± SE of relative gene expression from 4 to 8 independent experiments as indicated.

FIGURE S5 **(A,B)** RTPCR analysis of FBLN1C (gray bar) and FBLN1D (white bar) transcript levels in siRNA-mediated knockdown of FBLN1C (FBLN1Ci) and FBLN1D (FBLN1Di) in Calu-1 cells compared to their control (CON) (black bar) **(A)** cell adhesion and spreading (Figures 5A,B) **(B)** phosphorylated EGFR localization studies with serum growth factors (Figure 5C). Graph represents mean ± SE of relative gene expression from 3 to 4 independent experiments as indicated. **(C)** Western blot detection of Fibulin-1 (WB: FBLN1), Fibronectin (WB: FN), and GAPDH (WB: GAPDH) in lysates from Calu-1 cells in the presence of serum growth factors (5% FBS) in control (CON), FBLN1C (1Ci), FBLN1D (1Di) knockdown Calu-1 cells. Bar graphs represents mean ± SE of FBLN1/GAPDH and FN/GAPDH ratio from four independent experiments, normalized to respective control (CON). Statistical analysis of the data was done using the single sample *t*-test and *p* values are as shown.
