# Supplementary figures and images for: Cell Derived Matrix Fibulin-1 Associates With Epidermal Growth Factor Receptor to Inhibit Its Activation, Localization and Function in Lung Cancer Calu-1 Cells
Source: Front Cell Dev Biol. 2020 Jul 3;8:522. doi: 10.3389/fcell.2020.00522 (PMC7348071; doi:10.3389/fcell.2020.00522)

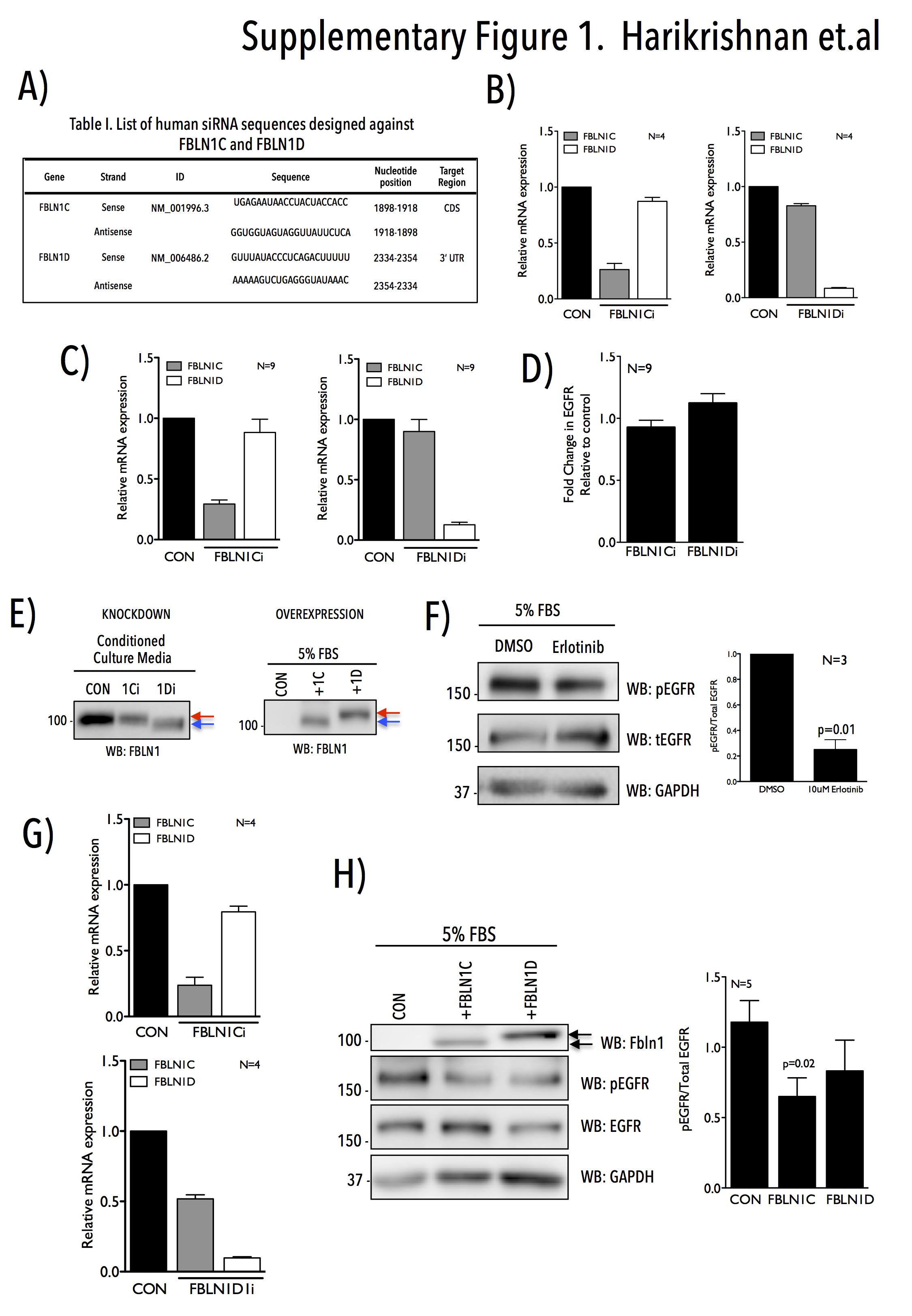

Supplement: FIGURE S1 — (A) Supplementary Table S1 lists custom designed siRNA sequences against FBLN1C and FBLN1D used for the knockdown experiments. (B) RTPCR analysis of FBLN1C (gray bar) and FBLN1D (white bar) transcript levels in siRNA-mediated knockdown of FBLN1C (FBLN1Ci) and FBLN1D (FBLN1Di) in Calu-1 cells compared to their control (CON) (black bar) (Figures 1F,G). Delta Delta Ct calculated relative to control was used to determine gene expression. Graph represents mean ± SE of relative gene expression from four independent experiments. (C,D) RTPCR analysis of FBLN1C (gray bar) (B), FBLN1D (white bar) (B) and EGFR (black bar) (C) in siRNA-mediated knockdown of FBLN1C (FBLN1Ci) and FBLN1D (FBLN1Di) in Calu-1 cells compared to their control (CON) (black bar) (Figures 1H,I). Delta Delta Ct calculated relative to control (CON) was used to determine fold change in gene expression. Graph represents mean ± SE of relative gene expression from nine independent experiments. (E) Comparison of western blots for the detection of FBLN1 (WB: FBLN1) in conditioned culture medium of Control (CON), FBLN1C (Ci) and FBLN1D (Di) knockdown cells (adapted from Figure 1F) and whole cell lysates of Calu-1 cells overexpressing FBLN1C (+1C) and FBLN1D (+1D) in the presence of serum (5% FBS) (adapted from Figure 1I). Blue and red arrows mark FBLN1C and FBLN1D, respectively. Images are representative of four independent experiments. (F) Western blot detection of EGFR phosphorylated on tyrosine 1173 (WB: pEGFR), total EGFR (WB: tEGFR) and GAPDH (WB: GAPDH) in Calu-1 cells grown with serum growth factors (5% FBS) and treated with DMSO or 10 μM Erlotinib. Bar graphs represents mean ± SE of pEGFR to total EGFR ratio from three independent experiments. Statistical analysis of the data was done using the one sample t-test and p values are as shown. (G) RTPCR analysis of FBLN1C (gray bar) and FBLN1D (white bar) transcript levels in siRNA-mediated knockdown of FBLN1C (FBLN1Ci) and FBLN1D (FBLN1Di) compared to control [file Image_1.TIFF]

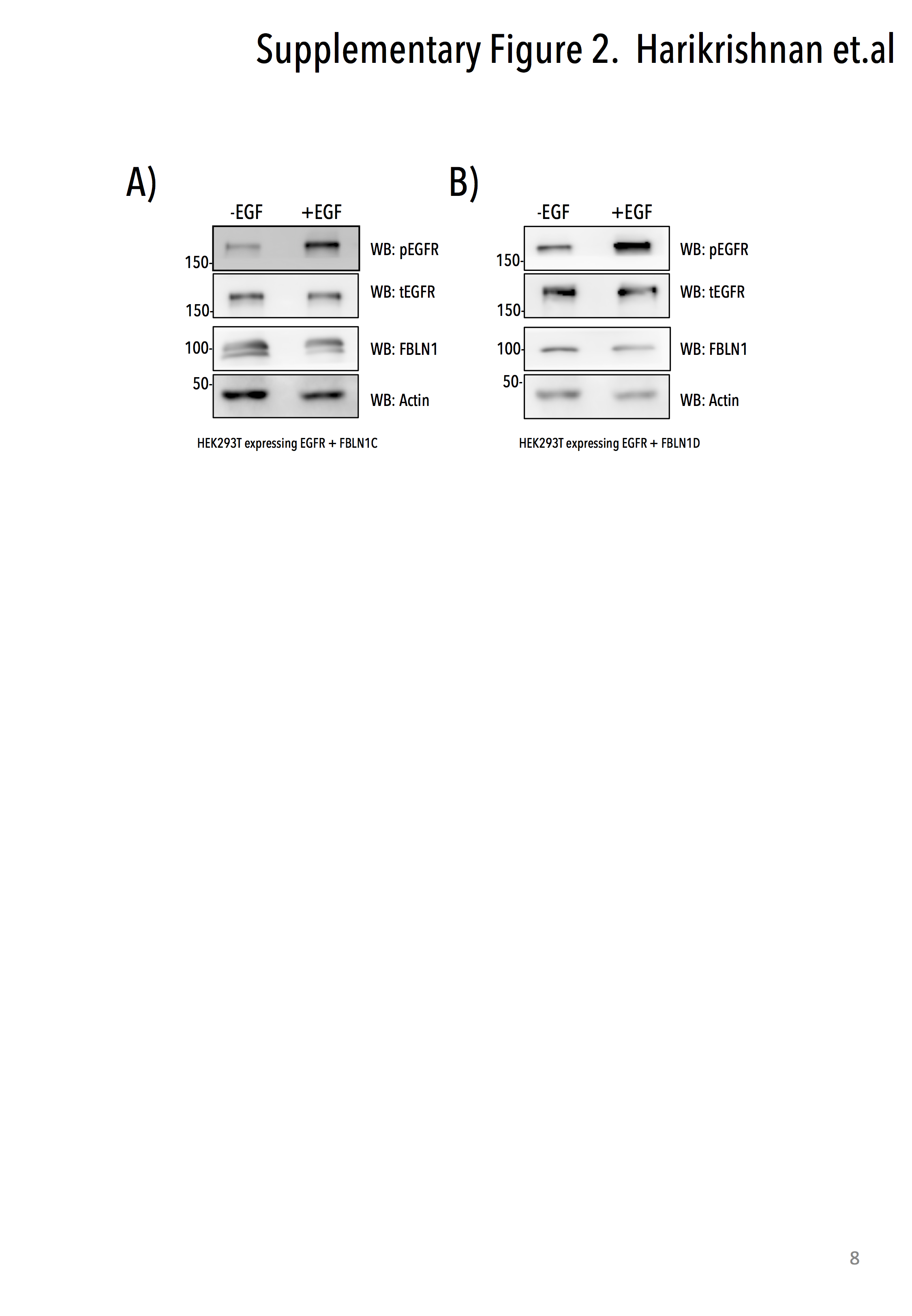

Supplement: FIGURE S2 — (A,B) Western blot detection of EGFR phosphorylated on tyrosine1173 (WB: pEGFR), total EGFR (WB: tEGFR), Fibulin-1 (WB: FBLN1), and Actin (WB: Actin) in lysates from serum deprived HEK293T cells (A) overexpressing EGFR-GFP and untagged FBLN1C or (B) EGFR-GFP and untagged FBLN1D and treated without (-EGF) or with stimulation using EGF (100 ng/ml) for 5 min (+EGF). Data is representative of three independent experiments with similar results. [file Image_2.TIFF]

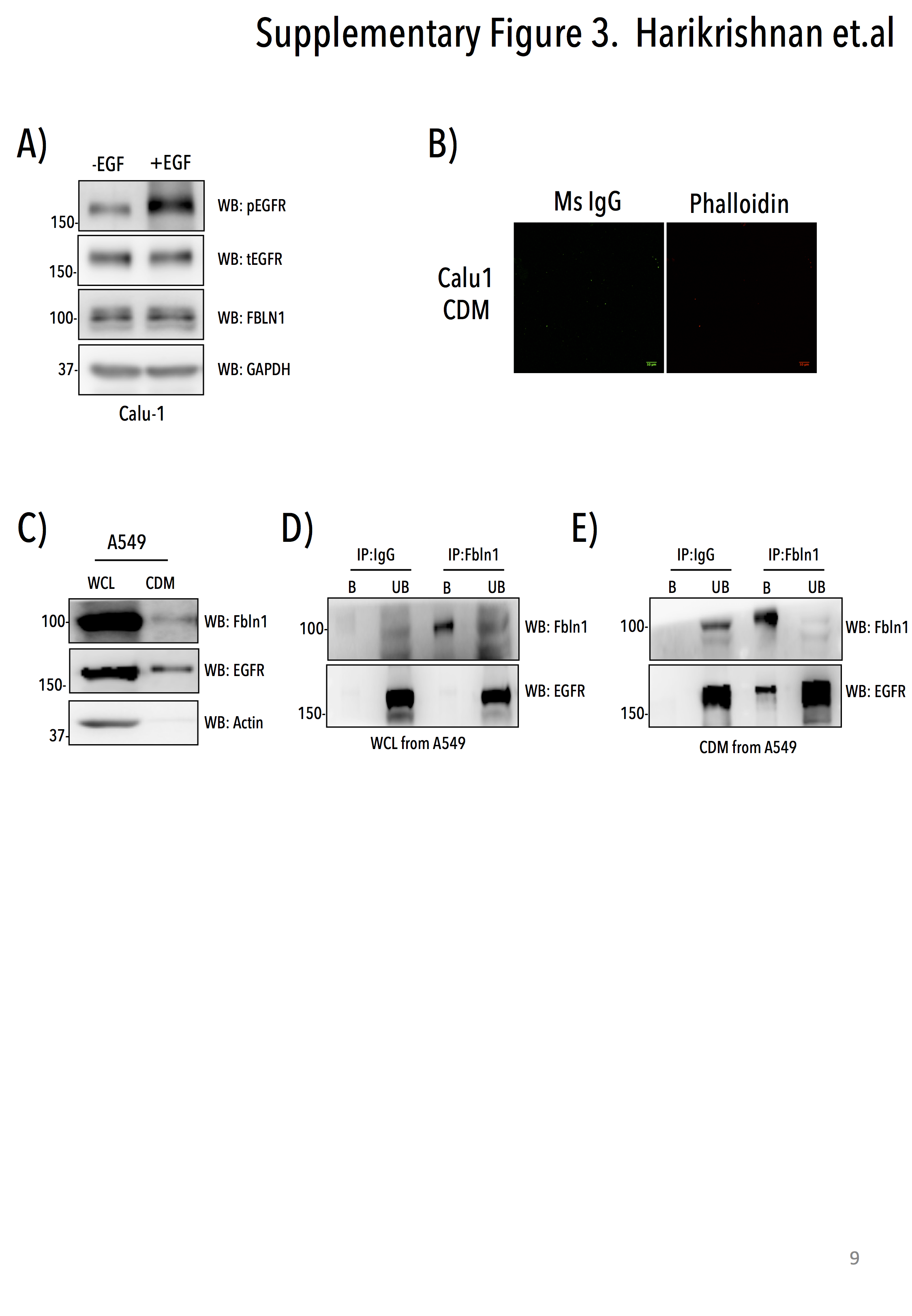

Supplement: FIGURE S3 — (A) Western blot detection of EGFR phosphorylated on tyrosine1173 (WB: pEGFR), total EGFR (WB: tEGFR), Fibulin-1 (WB: FBLN1) and GAPDH (WB: GAPDH) in lysates from serum deprived Calu-1 cells without (-EGF) on with stimulation using EGF (100 ng/ml) for 5 min (+EGF). Data is representative of three independent experiments with similar results. (B) CDM from Calu-1 cells fixed and immunostained using Alexa 488 conjugated mouse IgG (MsIgG) and phalloidin alexa-594 (Phalloidin). Representative confocal images are representative of three independent experiments with similar results. Scale bar represents 10 μm. (C) 10 μg of whole cell lysate (WCL) and cell derived matrix (CDM) from A549 cells were probed for Fibulin-1 (WB: FBLN1), EGFR (WB: EGFR), and Actin (WB: Actin) by western blot. The results are representative of three independent experiments. (D,E) Endogenous Fibulin-1 from (D) WCL, Fibulin-1 from CDM (E) of A549 cells was immunoprecipitated (IP: FBLN1) and compared to mouse IgG (IP: mIgG). Immunoprecipitation of Fibulin-1 (WB: FBLN1) and co-precipitation of EGFR (WB: EGFR) was tested by western blot. The immunoprecipitated (bound) protein eluted and un-bound fractions (B vs. UB) were also compared by western blot. The results are representative of three independent experiments which gave similar results. [file Image_3.TIFF]

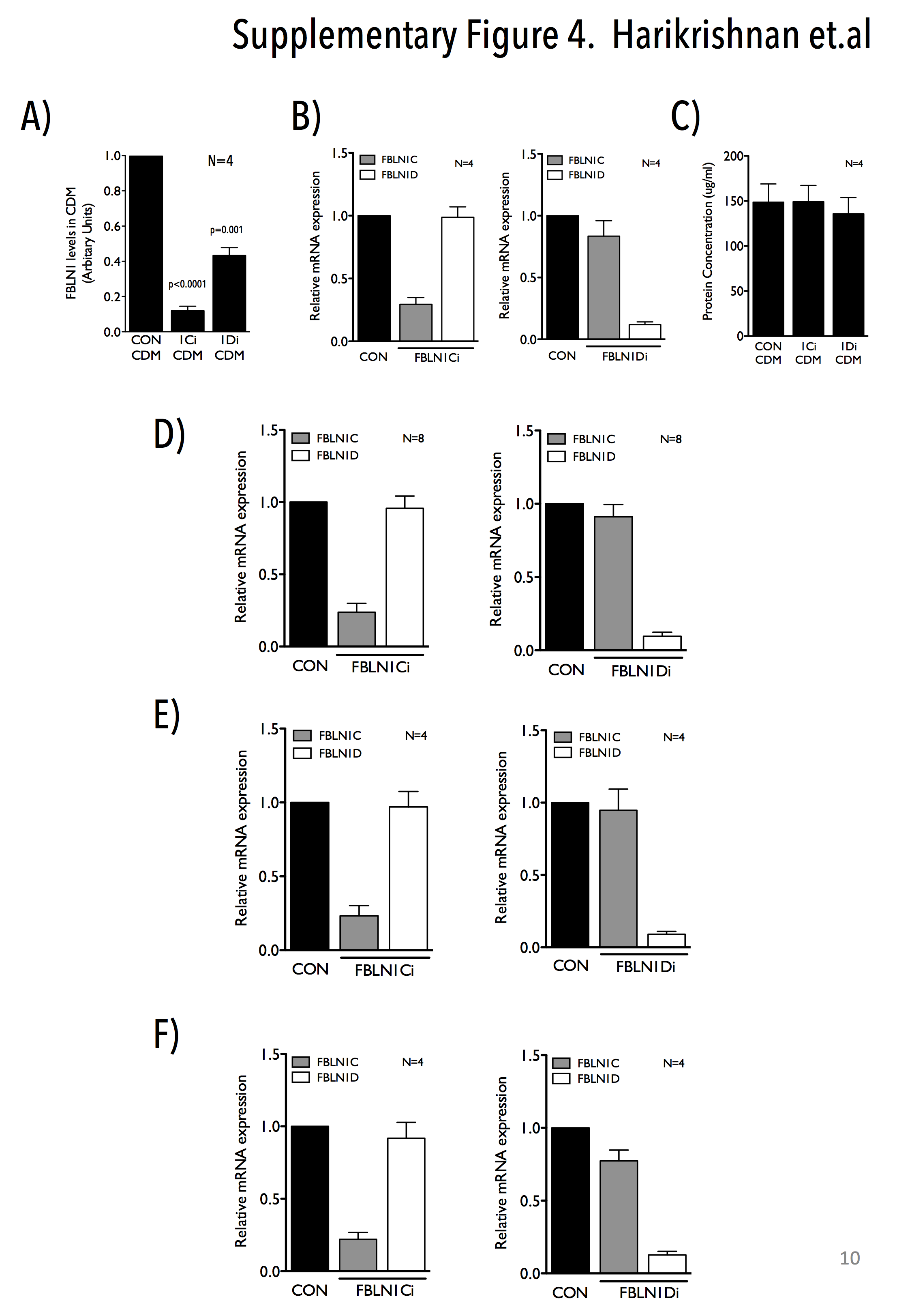

Supplement: FIGURE S4 — (A) Bar graphs represent mean ± SE of FBLN1 levels in CDM from 4 independent experiments normalized to control (CON). Statistical analysis of the data was done using the single sample t-test and p values are as shown. (B) RTPCR analysis of FBLN1C (gray bar) and FBLN1D (white bar) transcript levels in siRNA-mediated knockdown of FBLN1C (FBLN1Ci) and FBLN1D (FBLN1Di) in Calu-1 cells compared to their control (CON) (black bar). Delta Delta Ct calculated relative to control was used to determine gene expression. Graph represents mean ± SE of relative gene expression from four independent experiments as indicated. (C) Bar graphs represent mean ± SE of protein concentration in CDM quantified using BCA from four independent experiments as indicated. (D–F) RTPCR analysis of FBLN1C (gray bar) and FBLN1D (white bar) transcript levels in siRNA-mediated knockdown of FBLN1C (FBLN1Ci) and FBLN1D (FBLN1Di) in Calu-1 cells compared to their control (CON) (black bar) (D) in single cell migration (Figure 4C) (E) in EGFR activation studies with serum growth factors (Figure 4D) and (F) without serum growth factors (Figure 4E). Graph represents mean ± SE of relative gene expression from 4 to 8 independent experiments as indicated. [file Image_4.TIFF]

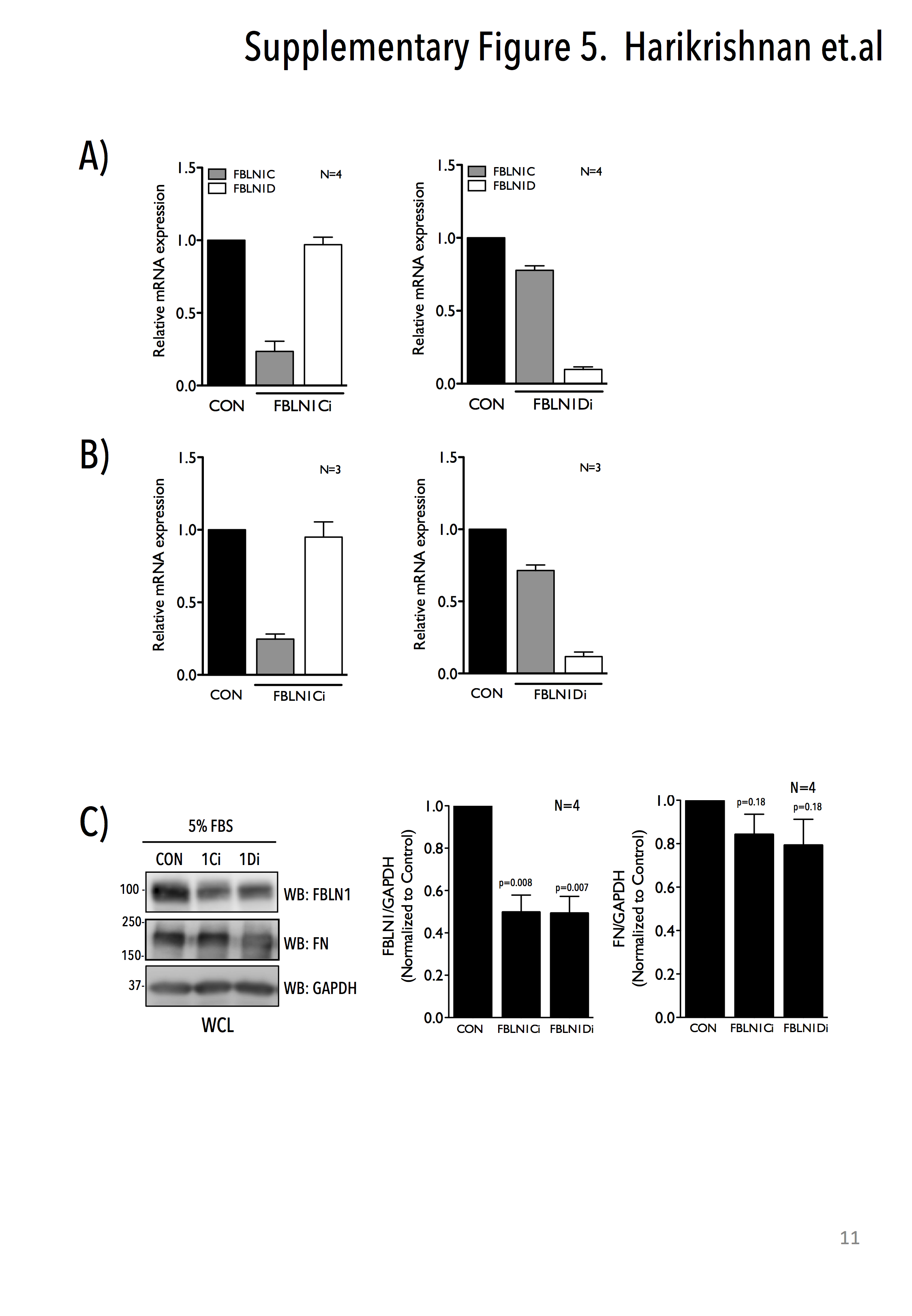

Supplement: FIGURE S5 — (A,B) RTPCR analysis of FBLN1C (gray bar) and FBLN1D (white bar) transcript levels in siRNA-mediated knockdown of FBLN1C (FBLN1Ci) and FBLN1D (FBLN1Di) in Calu-1 cells compared to their control (CON) (black bar) (A) cell adhesion and spreading (Figures 5A,B) (B) phosphorylated EGFR localization studies with serum growth factors (Figure 5C). Graph represents mean ± SE of relative gene expression from 3 to 4 independent experiments as indicated. (C) Western blot detection of Fibulin-1 (WB: FBLN1), Fibronectin (WB: FN), and GAPDH (WB: GAPDH) in lysates from Calu-1 cells in the presence of serum growth factors (5% FBS) in control (CON), FBLN1C (1Ci), FBLN1D (1Di) knockdown Calu-1 cells. Bar graphs represents mean ± SE of FBLN1/GAPDH and FN/GAPDH ratio from four independent experiments, normalized to respective control (CON). Statistical analysis of the data was done using the single sample t-test and p values are as shown. [file Image_5.TIFF]
